# Infraorbital Ethmoid Cells (Haller Cells) and Their Role in Chronic Rhinosinusitis: An Evidence-Based Meta-Analysis

**DOI:** 10.3390/life16071089

**Published:** 2026-06-29

**Authors:** Alin Horatiu Nedelcu, Emil Anton, Maria-Bianca Andrei, Otilia Elena Frăsinariu, Anton Knieling, Ancuta Lupu, Razvan Tudor Tepordei, Simona Alice Partene Vicoleanu, Gabriel Statescu, Manuela Ursaru, Daniela Carmen Rusu, Sorana Caterina Anton, Ionela Daniela Morariu, Mihaela Mitrea, Vasile Valeriu Lupu

**Affiliations:** 1 Grigore T. Popa University of Medicine and Pharmacy Iasi, 16 Universitatii Street, 700115 Iasi, Romania; alin.nedelcu@umfiasi.ro (A.H.N.); emil.anton@umfiasi.ro (E.A.); bianca_maria.2003@yahoo.com (M.-B.A.); otiliafrasinariu@gmail.com (O.E.F.); tony_knieling@yahoo.com (A.K.); razvan.tepordei@umfiasi.ro (R.T.T.); partene.vicoleanu@umfiasi.ro (S.A.P.V.); gabriel.statescu@umfiasi.ro (G.S.); manuela.ursaru@umfiasi.ro (M.U.); danarusu2005@yahoo.com (D.C.R.); sorana.anton@umfiasi.ro (S.C.A.); ionela.morariu@umfiasi.ro (I.D.M.); mihaela.mitrea77@yahoo.com (M.M.); vasile.lupu@umfiasi.ro (V.V.L.); 2 Radiology Clinic, Clinical Rehabilitation Hospital Iasi, 700661 Iasi, Romania; 3 Radiology Clinic, “Sf Spiridon” County Clinical Emergency Hospital, 700111 Iasi, Romania

**Keywords:** Haller cells, infraorbital ethmoid cells, maxillo-ethmoidal cells, chronic rhinosinusitis, pneumatized cells, maxillary sinus, maxillary infundibulum

## Abstract

Background: Haller cells (HCs), infraorbital pneumatization of the anterior ethmoidal air cells, are anatomical variants frequently located adjacent to the maxillary sinus ostium. Their presence has been hypothesized to interfere with mucociliary drainage and contribute to the pathophysiology of chronic rhinosinusitis (CRS). Despite numerous imaging-based investigations, the etiological role of Haller cells in CRS remains uncertain. The aim of this meta-analysis is to highlight the association between HC and CRS using data derived from Computed Tomography (CT) and Cone-Beam Computed Tomography (CBCT) imaging. Materials and Methods: Following PRISMA guidelines and a registered protocol in the PROSPERO database, a systematic search across PubMed, EMBASE, and COCHRANE databases led to the inclusion of eight studies published between 2011 and 2024, comprising 2011 patients. The included studies employed multiplane reconstruction techniques and high-resolution imaging protocols. The majority of studies were retrospective, with only one adopting a prospective design. Results: The pooled odds ratio (OR) for the association between HC and CRS was 1.54 (95% Confidence Interval: 0.93–2.54; *p* = 0.09), suggesting a non-significant trend toward a positive correlation. Heterogeneity was substantial (I^2^ = 87%, *p* < 0.01), due to differences in study design, populations, and diagnostic criteria, contributing to inconsistency in reported estimates across the studies. Moreover, Egger’s test showed no evidence of publication bias (intercept = 0.67, *p* = 0.895). Although three studies fulfilled all eleven evaluation criteria outlined by the JBI (Joanna Briggs Institute) checklist, the observed variability limits the interpretability of pooled outcomes. Conclusions: These findings suggest that, while HC may play a role in CRS pathogenesis, the current evidence does not support a statistically significant association. Future prospective research using standardized imaging and diagnostic criteria is essential to clarify the clinical impact of HC in sinonasal disease.

## 1. Introduction

Swiss anatomist Albert von Haller initially reported in 1765 the discovery of Haller’s cells, usually named infraorbital ethmoid or maxillo-ethmoidal cells. Often located under the ethmoid bulla and next to the lamina papyracea, these are pneumatized extensions of the anterior ethmoid sinus that are situated near the orbital floor and the superior face of the maxillary sinus [1,2,3,4,5]. From an embryological point of view, it is thought that these cells grow during the ethmoid sinus pneumatization phase [3,5,6]. The Haller’s cells can appear in the superior part of the maxillary sinus as a result of pneumatization of the lateral aspect of the ethmoid bone during embryonic development [3]. About 40% of people present this variant, which represents the result of the variations in how the paranasal sinuses develop [5]. The presence of Haller’s cells in embryos can cause different clinical repercussions. The size and location of these cells can make people more susceptible to symptoms including sinusitis, nasal blockage, and facial pain because they can interfere with normal mucociliary flow and aggravate maxillary sinusitis [3,4,5,6]. Regardless of being frequently overlooked, Haller’s cells are important for sinus health because they can determine constriction and obstruction in the maxillary sinus ostium and the ethmoidal infundibulum due to their closeness to these structures [3,4]. According to studies, these cells are statistically more relevant in developing exacerbations of chronic rhinosinusitis than the other structural modifications, such as septal deviation or concha bullosa [7,8,9,10,11,12]. Haller’s cells can be identified on imaging due to their precise position, commonly appearing as coincidental discoveries on panoramic radiography or Computed Tomography (CT) scans [4,13]. In order to decrease intraoperative risks, doctors should take these anatomical and embryological variants into consideration when planning surgeries, as radiographic studies show a correlation between sinus problems and the size of Haller’s cells, which should be <3 mm [5]. The necessity of a precise anatomical evaluation in endonasal treatments to prevent intraoperative risks is highlighted by the fact that these cells make the surgical access to the maxillary sinus or anterior ethmoid cells very difficult [3,13].

Rhinosinusitis is an inflammatory disease that affects the sinonasal mucosa, affecting a variety of age groups. From a radiological standpoint, mucosal thickening greater than 2 mm is generally considered a pathological finding, reflecting chronic inflammatory involvement of the sinonasal mucosa. This thickening may be focal or diffuse and is most commonly assessed using computed tomography, which remains the gold standard imaging modality for the evaluation of paranasal sinus pathology. In addition, sclerosis of the sinus walls represents a secondary sign of long-standing inflammation. It is characterized by increased bone density and thickening of the sinus walls, resulting from chronic osteitic changes. These alterations suggest a prolonged disease course and may be associated with impaired mucociliary clearance and persistent obstruction of sinus drainage pathways. While the episodes of acute rhinosinusitis (ARS) in children are seen annually, the prevalence of pediatric cases, especially of chronic rhinosinusitis (CRS), varies between 5% and 15%, depending on the country [14]. The symptoms for both rhinosinusitis and other illnesses, such as allergic rhinitis, adenoiditis, or colds, overlap and change with age, making it difficult to differentiate between them. For example, older children usually present nasal stuffiness, while younger children frequently show coughs and colored discharge [14]. Approximately 11% of the population from Europe presents CRS, while in the United States, the percentage is higher, 16%, and lower percentages are seen in the regions of Asia or South America [15]. Chronic rhinosinusitis (CRS) is traditionally classified into two phenotypic subtypes based on the presence or absence of nasal polyps: CRS with nasal polyps (CRSwNP) and CRS without nasal polyps (CRSsNP). From an endotypic perspective, CRS is further categorized into three principal endotypes (Type 1, Type 2, and Type 3), each characterized by distinct inflammatory pathways and gene expression profiles. In clinical practice, a simplified and widely applied classification distinguishes between Type 2 CRS and non-Type 2 CRS, depending on the presence or absence of Type 2 inflammation. This endotype-based stratification holds significant clinical relevance, particularly in guiding personalized therapeutic decisions [15,16,17,18,19,20]. Comorbidities like exacerbated respiratory disease (AERD) or asthma can also be used for CRS classification [15,16,17]. European Standards (EP3OS) have recently established defined criteria to help with research and to improve diagnosis; however, there is still a lack of data on CRS prevalence in developing countries despite high direct medical expenses [21,22].

Given the frequency and clinical impact of Haller’s cells and other anatomical variations in recurrent and chronic rhinosinusitis, these structures should be carefully evaluated in the context of diagnosing and planning treatment for sinonasal conditions. Such an evaluation not only helps to avoid intraoperative risks but also contributes to a deeper understanding of how anatomical factors influence sinus health [23].

Our study aims to critically analyze the association between Haller cells and chronic rhinosinusitis identified through CT imaging. The key questions we seek to answer are: Is the presence of Haller cells an etiological factor in maxillary chronic rhinosinusitis?

## 2. Materials and Methods

The methodology for conducting this systematic review followed the PRISMA guidelines (http://www.prisma-statement.org/ (accessed on 1 May 2026)). The study protocol, analysis method, and inclusion and exclusion criteria were pre-defined and documented in a protocol to reduce the risk of post hoc selective bias. (PROSPERO code: CRD420250653748).

### 2.1. PICO

Population: Clinical studies involving patients aged 18 and older that investigated the relationship between Haller cells and maxillary chronic rhinosinusitis.

Intervention: Three-dimensional evaluation of upper airway and craniofacial morphology using a CT or CBCT scanner.

Comparison: Assessing the relationship between the presence of Haller cells and chronic rhinosinusitis. The impact of Haller cells on the morphology and morphometry of the maxillary sinus infundibulum.

Outcome: Identifying Haller cells as an etiological factor in chronic maxillary rhinosinusitis.

### 2.2. Literature Search Strategy

The protocol for this systematic review was registered in the International Register of Systematic Reviews—PROSPERO code: CRD420250653748.

**Figure 1:**
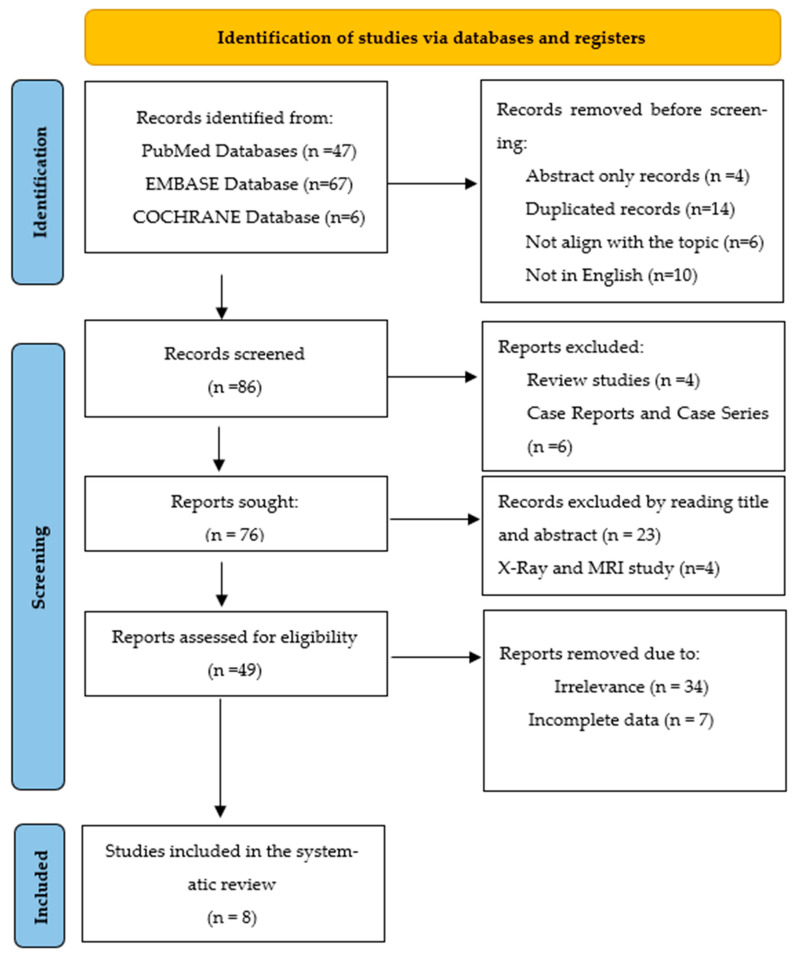
PRISMA chart flow—literature search strategy.

Two independent authors conducted the literature search in PubMed, EMBASE and COCHRANE databases: We initiated the search protocol on 2 December 2024 for articles published between January 1991 and December 2024. The PubMed search used the following keywords: (“Haller cells”[All Fields] OR “Haller’s cells”[All Fields] OR “infraorbital cells”[All Fields] OR “orbitoethmoidal”[All Fields]) AND (“chronic rhinosinusitis”[All Fields] OR “chronic sinusitis”[All Fields] OR “clinical”[All Fields]). The search query for EMBASE was set as follows: (‘Haller cells’ OR ‘infraorbital cells’) AND (‘rhinosinusitis’ OR ‘clinical’). We applied the search formula (“Haller cells” OR “Haller’s cells”) AND (“chronic rhinosinusitis” OR “chronic sinusitis” OR “clinical”) in the Cochrane Database, and after running it, we retrieved six articles. After reviewing the abstracts, we selected six articles from PubMed (out of 47) and two articles from EMBASE (out of 67). Manuscripts from the COCHRANE database (6 studies) were excluded, with two being duplicates and four not aligning with the topic. After applying the inclusion and exclusion criteria (Table 1), eight articles were retained for this review. The reference lists of the selected articles were reviewed manually to identify any publications that may have been overlooked in the database searches. A third investigator, who had the final say, resolved any disagreements between the investigators regarding article selection. The search strategy is illustrated in a flow chart (Figure 1) in accordance with PRISMA guidelines [24].

### 2.3. Statistical Analysis

All statistical analyses were conducted using Microsoft Excel and the dedicated tools available through MetaAnalysisOnline.com, an online platform designed for meta-analytical computations. For each of the studies included in the final review, the odds ratio (OR) was calculated, along with the corresponding 95% confidence interval (CI)—specifically, both the lower and upper confidence limits were determined to assess the precision and reliability of the effect estimates.

To assess statistical heterogeneity among the included studies, we employed two commonly used tests:

- The Tau^2^ (Tau-squared) test, which estimates the between-study variance.
- The I^2^ (I-squared) statistic, which quantifies the percentage of total variation across studies that is due to heterogeneity rather than chance.

Standard thresholds guided the interpretation of I^2^ values, with higher values indicating greater heterogeneity.

The overall effect size across all studies was evaluated using the Z-test, which tests the null hypothesis that the pooled effect is equal to zero. A *p*-value of less than 0.05 was considered statistically significant.

In order to assess the presence of publication bias, a funnel plot was constructed. This visual tool allows for the evaluation of asymmetry in the distribution of study results, which can suggest potential bias in the published literature. The level of statistical significance for all tests, including assessments of heterogeneity and overall effect, was set at *p* = 0.05.

## 3. Results

### 3.1. Database Search Results

Following a standardized and systematic search across three major databases—PubMed, EMBASE, and the Cochrane Library—120 articles were initially identified. These records were screened according to predefined inclusion and exclusion criteria established in the study protocol. After title and abstract screening, followed by full-text review, 112 articles were excluded for various reasons, including lack of relevance to the research question, duplication, or failure to meet methodological requirements. Ultimately, eight full-text studies were deemed eligible and included in the final analysis. These selected studies were published between 2011 and 2024, reflecting the most recent and relevant contributions to the topic under investigation. The selection process is illustrated in Figure 1.

### 3.2. Study Characteristics

The present meta-analysis incorporates eight studies, comprising both prospective and retrospective research designs. Of these, one study—conducted by Nandyal et al. [25]—was designed prospectively, while the remaining seven studies followed a retrospective approach.

An analysis of the ethno-geographic distribution of the included studies reveals a predominant representation of Asian populations. Specifically, four studies were conducted in India [25,26,27,28], while three were from Turkey [29], Hong Kong [30], and Malaysia [31], respectively. In contrast, only one study—conducted by Nedelcu et al. [12] in Romania—focused on a Caucasian population. This distribution highlights a potential regional bias in the current literature and should be considered when interpreting the generalizability of the meta-analysis findings.

In terms of sample size, the cumulative number of patients across all included studies totaled 2011 individuals, with an age range spanning from 16 to 85 years. The gender distribution was relatively balanced, consisting of 951 male and 1060 female participants.

With respect to imaging modalities, a variety of techniques were employed across the included studies. Specifically:

Three studies utilized conventional computed tomography (CT) [25,28,31].

One study applied multi-detector computed tomography (MDCT) [12].

Four studies implemented cone-beam computed tomography (CBCT) [26,27,29,30].

Regarding image acquisition protocols, five out of the eight studies reported the use of thin-slice imaging, with slice thicknesses ranging from 0.1 mm to 0.6 mm, thereby ensuring high-resolution anatomical detail. The remaining three studies did not provide specific information regarding the slice thickness used during image acquisition.

Importantly, multiplanar reconstruction (MPR) techniques were employed in all eight studies, allowing for detailed visualization and comprehensive assessment of anatomical structures in multiple planes.

These demographic and methodological details provide important context for interpreting the findings of the meta-analysis and assessing the generalizability of the results (Table 2).

### 3.3. Study Quality Assessment

The methodological quality of the studies included in this meta-analysis was rigorously assessed using the Critical Appraisal tools provided by the Joanna Briggs Institute (JBI) specifically for systematic reviews and meta-analyses. This evaluation involved a comprehensive examination of each of the eight included studies against eleven distinct quality criteria. For each criterion, a binary judgment was made: the criterion was deemed “met” (indicated by “x”) if the study adequately addressed the methodological requirement, or “not met” (indicated by “0”) if it did not (Table 3).

### 3.4. Evaluation Indexes of Diagnostic Tests

**Figure 2:**
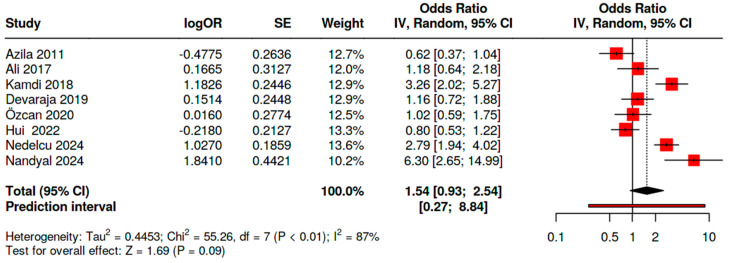
Forest plot summarizing the meta-analysis of the relationship between Haller cells and chronic rhinosinusitis. (Azila 2011 [31], Ali 2017 [26], Kamdi 2018 [27], Devaraja 2019 [28], Özcan 2021 [29], Hui 2022 [30], Nedelcu 2024 [12], Nandyal 2024 [25]).

A total of eight studies were included in the meta-analysis. Using a random effects model with the inverse variance method, the pooled odds ratio (OR) for the association between Haller cells and chronic rhinosinusitis (CRS) was calculated as 1.54, with a 95% confidence interval (CI) of 0.93 to 2.54. Although the OR indicates a possible positive association, the confidence interval includes the value 1.0, suggesting that the result is not statistically significant (Figure 2).

The test for overall effect did not reach statistical significance, indicating that no conclusive association could be confirmed across the included studies.

Moreover, a high level of heterogeneity was observed (I^2^ = 87%, *p* < 0.01), implying substantial variability in the effect estimates among the studies. This suggests that differences in study design, populations, or diagnostic criteria may have influenced the results and contributed to the lack of consistency across studies.

### 3.5. Publication Bias Assessment

**Figure 3:**
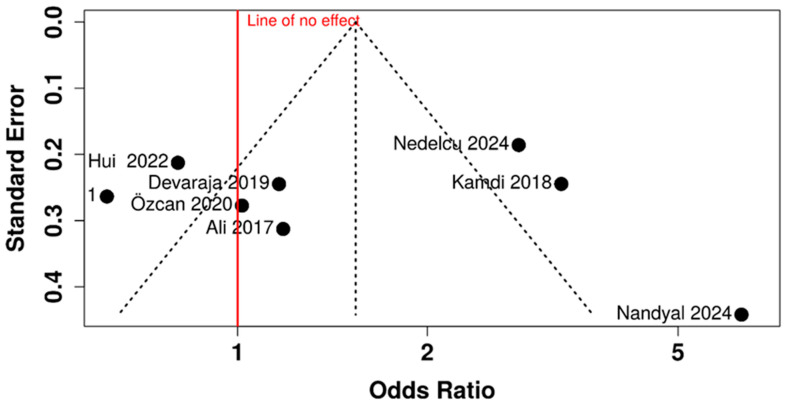
The funnel plot assessing potential publication bias. (Azila 2011 [31], Ali 2017 [26], Kamdi 2018 [27], Devaraja 2019 [28], Özcan 2021 [29], Hui 2022 [30], Nedelcu 2024 [12], Nandyal 2024 [25]).

The assessment of potential publication bias in this meta-analysis, using both visual inspection of the funnel plot and the statistical Egger’s test, suggests that publication bias is unlikely to be a significant factor influencing the overall findings (Figure 3). The plot’s symmetry is further examined through Egger’s test, the results of which are provided within the figure’s caption. The intercept from Egger’s regression is 0.67, with a 95% confidence interval ranging from −8.86 to 10.2. The calculated t-statistic is 0.137, yielding a corresponding *p*-value of 0.895.

## 4. Discussion

The relationship between anatomical variants of pneumatized facial structures and chronic rhinosinusitis (CRS) represents a topic of considerable clinical relevance, particularly due to the high incidence of sinonasal disorders encountered in daily practice. Among these anatomical variants, Haller cells—ethmoidal air cells located inferior to the orbit and adjacent to the maxillary sinus infundibulum—are of particular interest. Their close proximity to key drainage pathways can result in partial obstruction of the osteomeatal complex. This anatomical positioning may predispose patients to impaired mucociliary clearance, leading to retention of secretions within the maxillary sinus and fostering an environment conducive to recurrent or chronic infection.

In this context, Haller cells are increasingly recognized as potential contributing factors to the development and persistence of CRS. Although the presence of these cells does not necessarily indicate pathology, their size, degree of pneumatization, and spatial orientation relative to adjacent structures can influence their clinical significance.

To better understand this association, we conducted a comprehensive meta-analysis including eight clinical studies, encompassing both prospective and retrospective designs—one prospective and seven retrospectives. Collectively, these studies evaluated a total of 2011 patients, making this, to the best of our knowledge, the largest meta-analysis to date focused exclusively on the potential correlation between Haller cells and chronic rhinosinusitis. This robust sample size and the diversity of imaging modalities included lend credibility to the findings and provide a valuable synthesis of the current state of evidence.

A significant observation from the quality assessment was that three of the eight included studies, namely, references [12,27,29], successfully fulfilled all eleven evaluation criteria outlined by the JBI (Joanna Briggs Institute) checklist. This signifies a high level of methodological rigor in these particular studies, suggesting a robust design, execution, and reporting that minimizes the risk of bias and enhances the trustworthiness of their findings.

The overall methodological quality of the included studies was further quantified by calculating an average methodological score of 9.75. This score represents the mean number of quality criteria met across the eight studies, with individual study scores ranging from a minimum of 8 to a perfect maximum of 11 met criteria. This high average score, coupled with the fact that three studies achieved a perfect score, suggests a generally strong methodological foundation for the evidence base synthesized in this meta-analysis.

Based on these scores, the majority of the studies, specifically six out of the eight, were classified as “high quality”. This categorization indicates that these studies adhered to a significant number of the JBI’s quality criteria, implying a lower susceptibility to methodological flaws that could potentially compromise the validity of their results. The remaining two studies, identified as [28,31], were classified as meeting a “moderate quality” standard. However, the text emphasizes that the methodological scores of these two studies were very close in value to the threshold for the “high quality” classification, suggesting that their methodological limitations were likely minor and did not substantially detract from their overall contribution to the evidence base.

The generally high methodological quality of the included studies, with a significant portion classified as “high quality” and a high average score, strengthens the foundation for the evidence synthesis in this meta-analysis. This suggests that the findings of the meta-analysis are likely to be based on reliable and trustworthy individual studies.

The fact that three studies fully met all evaluation criteria provides a core of particularly robust evidence. The high average score and the classification of the majority of studies as “high quality” increase the confidence in the overall findings and conclusions of the meta-analysis.

The identification of several high-quality studies contributes significantly to the body of knowledge in this field. These studies can serve as examples of rigorous research methodology for future investigations.

In conclusion, the methodological quality assessment of the included studies using JBI criteria indicates a generally strong evidence base for this meta-analysis. The high average score, the presence of three studies meeting all criteria, and the classification of the majority as “high quality” suggest that the findings of this review are likely to be robust. The two “moderate quality” studies, with scores close to the “high quality” threshold, likely pose a minimal threat to the overall conclusions. This strong methodological foundation enhances confidence in the synthesized evidence and its potential to inform practice and future research.

The results of the meta-analysis, as visually represented in the Forest plot (Figure 2), suggest a trend toward a possible association between the presence of Haller cells and chronic rhinosinusitis (CRS), with a pooled odds ratio (OR) of 1.54. However, the 95% confidence interval (0.93–2.54) includes the null value, indicating that this association does not reach statistical significance. Therefore, based on the aggregated data, a definitive conclusion regarding the role of Haller cells as a risk factor for CRS cannot be drawn.

From a clinical perspective, these findings should be interpreted with caution. Although Haller cells cannot currently be considered an independent or validated risk factor for CRS, the observed trend suggests a potential contributory role, particularly in specific anatomical or functional contexts. Given their location adjacent to the maxillary infundibulum, Haller cells may influence sinus drainage pathways and mucociliary clearance, potentially predisposing to localized obstruction in susceptible individuals. Therefore, while the presence of Haller cells alone should not be used as a predictive marker for CRS, their identification remains clinically relevant in imaging evaluation, especially in patients with unexplained or refractory symptoms. In such cases, they may act as an adjunctive anatomical factor that contributes to disease expression rather than a primary etiological determinant. In summary, the clinical importance of these findings lies not in establishing causality but in supporting a multifactorial model of CRS, where anatomical variations such as Haller cells may modulate disease risk in conjunction with other inflammatory, environmental, and host-related factors.

In addition, the analysis revealed a high degree of heterogeneity among the included studies (I^2^ = 87%, *p* < 0.01), suggesting that the variability in results is largely due to true differences in study populations, methodologies, or diagnostic criteria rather than random chance. This significant heterogeneity reduces the certainty of the pooled estimate and highlights the need for careful interpretation. Several plausible sources of heterogeneity can be identified.

From a methodological perspective, differences in study design (retrospective vs. prospective), sample size, and inclusion criteria may significantly influence the reported outcomes. Studies with small sample sizes are more prone to variability and may disproportionately affect pooled estimates. A major contributor is likely the lack of standardization in imaging protocols and diagnostic criteria. Variability in CT acquisition parameters, slice thickness, and interpretation criteria for identifying Haller cells may lead to inconsistent detection rates. Additionally, differences in defining chronic rhinosinusitis (CRS), whether based on imaging findings alone or combined clinical criteria, can further amplify heterogeneity.

Secondly, population-related factors also play an important role. Differences in age distribution, ethnic background, environmental exposures, and comorbidities (such as allergy or asthma) may influence both the prevalence of Haller cells and susceptibility to CRS.

Another key source is observer variability, as the identification of anatomical variants such as Haller cells is, to some extent, operator-dependent. Studies involving different levels of radiological expertise or lacking interobserver agreement analysis may introduce inconsistency.

In summary, the high heterogeneity reflects the complex and multifactorial nature of CRS and the variability in how anatomical variants are assessed across studies. Addressing these sources through methodological standardization and more rigorous study design will be essential for clarifying the true clinical significance of Haller cells.

The lack of statistical significance, despite a suggestive point estimate, combined with the observed heterogeneity, points to the necessity for further, more homogeneous studies, preferably prospective in design, with standardized imaging protocols and well-defined criteria for anatomical classification and CRS diagnosis. Such studies would be essential to clarify the potential clinical impact of Haller cells on sinus drainage and inflammation.

The funnel plot did not visually indicate any substantial asymmetry. However, the symmetrical appearance of the funnel plot in this meta-analysis implies that the included studies are likely a representative sample of the available research, irrespective of their statistical significance or direction of effect.

Egger’s test is a statistical test designed to formally assess the asymmetry of the funnel plot. A statistically significant *p*-value (typically <0.05) from Egger’s test would suggest the presence of funnel plot asymmetry, thereby raising concerns about potential publication bias. In this meta-analysis, Egger’s test yielded an intercept of 0.67 with a 95% confidence interval ranging from −8.86 to 10.2 and a corresponding t-statistic of 0.137 with a *p*-value of 0.895.

The non-significant *p*-value of 0.895 indicates that Egger’s test does not provide statistical evidence of funnel plot asymmetry. Furthermore, the 95% confidence interval for the intercept (−8.86 to 10.2) includes zero, which also supports the conclusion of no significant asymmetry. The intercept of Egger’s regression line quantifies the degree of asymmetry; an intercept significantly different from zero suggests asymmetry. In this case, the intercept of 0.67 is not statistically different from zero.

The findings from both the visual inspection of the funnel plot and the statistical Egger’s test provide reassuring evidence that publication bias is unlikely to have substantially distorted the results of this meta-analysis. This increases confidence that the pooled effect estimate is a reasonably unbiased representation of the available evidence regarding the association between Haller cells and chronic rhinosinusitis.

However, it is important to note that while these tests are valuable tools for assessing publication bias, they are not definitive proof of its absence. Funnel plot asymmetry can sometimes arise due to factors other than publication bias, such as genuine heterogeneity or small study effects. Conversely, publication bias might exist even in the absence of clear funnel plot asymmetry, particularly if the number of included studies is small. Given that this meta-analysis included eight studies, the power of these tests to detect subtle publication bias might be limited.

Despite these limitations, the current evidence from the funnel plot and Egger’s test suggests that the lack of a statistically significant pooled odds ratio is unlikely to be solely attributable to the selective publication of studies with statistically significant findings. The observed non-significant association and the high heterogeneity remain the primary findings to consider when interpreting the overall evidence.

In conclusion, while acknowledging the inherent limitations of publication bias assessment methods, the results of the funnel plot inspection and Egger’s test do not provide evidence of significant publication bias in this meta-analysis. This strengthens confidence in the reported pooled estimate as being less likely to be skewed by the selective publication of studies. However, the non-significant overall effect and the substantial heterogeneity across studies remain key aspects of the findings that warrant careful consideration.

## 5. Conclusions

The heterogeneity of results obtained could not confirm evidence towards a strict correlation between Haller cells and maxillary CRS. This seems to be justified by the lack of homogeneity between CT and CBCT protocols. For greater clarity and consistency in research on this topic, future studies ought to implement standardized imaging approaches and adhere to rigorous, well-defined criteria for both anatomical classifications and the diagnosis of CRS.

## Figures and Tables

**Table 1:** Inclusion and exclusion criteria.

Inclusion Criteria	Exclusion Criteria
1. Articles on the association between Haller cells and maxillary chronic rhinosinusitis	1. X-Ray and MRI studies
2. CT and CBCT studies	2. Studies that do not benefit from comparative analysis
3. Full text available	3. Only abstract available
4. Observational imaging studies	4. Reviews, Observational studies, Case Reports and Case Series, Conference articles
5. Manuscript in English	5. Studies not in English
6. Human over 12 years old studies	6. Studies involving non-human subjects.

**Table 2:** Study summary. CT—computed tomography; MDCT—multidetector computed tomography; CBCT—cone beam computed tomography; a/c/s—multiplanar reconstructions: axial/coronal/sagittal; HC—Haller cells; CRS—chronic rhinosinusitis.

Study	Year	Country	Study Type	Sample Size (Patients)	Age (Years)	Males	Females	Investigation	Thickness Reconstruction (mm)		Short Description of the Study	Outcome
Azila et al. [31]	2011	Malaysia	retrospective/group control	240	x	95	145	CT	a/c/s	1.25	Patients were divided into two groups: Group I included 120 subjects diagnosed with chronic rhinosinusitis (CRS), and Group II (control group) included 120 subjects without clinical signs of CRS. The association between rhinosinusal anatomical variations and CRS was assessed.	Infraorbital ethmoid cells were implicated as a possible etiologic factor in CRS due to their negative influence on maxillary sinus ventilation (*p* < 0.05)
Ali et al. [26]	2017	India	retrospective	201	16 to 85 years (mean, 37 years)	97	104	CBCT	a/c/s	x	The study evaluated the association of Haller cells and accessory maxillary ostia with chronic maxillary rhinosinusitis.	No statistical correlation was achieved between HC and maxillary CRS (*p* = 0.599)
Kamdi et al. [27]	2018	India	retrospective	200	16 to 73 years (mean, 32 years)	138	62	CBCT	a/c/s	0.4	The study evaluated the prevalence, morphology and location of Haller cells as well as the association with chronic maxillary rhinosinusitis.	HC correlates strongly with maxillary CRS (*p* = 0.000001)
Devaraja et al. [28]	2019	India	retrospective	151	18 to 71 years; mean 42 years	50	101	CT	a/c/s	0.6	Anatomical variation of paranasal sinuses and association with CRS	44 HC out of 106 maxillary CRS (*p* = 0.54)
Özcan et al. [29]	2021	Turkey	retrospective	621	18 to 64 years; mean 33.46	278	343	CBCT	a/c/s	0.1 to 0.3	Evaluation of the relationship of Haller cells with the maxillary ostium and the causal relationship with rhinosinusal pathology	21 out of 61 sinuses with HC have CRS (*p* = 0.95); prevalent HC—Female:Male ratio was 3:1 and it was found statistically significant (*p* = 0.004)
Hui et al. [30]	2022	Hong Kong	retrospective	273	>12 years	111	162	CBCT	a/c/s	0.4	Evaluation of the causal relationship between Haller cells and rhinosinusal pathology	50 out of 108 patients with Haller cells have maxillary CRS (*p* = 0.303)
Nedelcu et al. [12]	2024	Romania	randomized/retrospective	255	60.40 ± 18.37 years	140	115	MDCT	a/c/s	0.5	Comparative analysis of the association between Haller cells and chronic rhinosinusitis in two seasonal cohorts: “Summer” group vs. “Winter” group	HC is a predisposing factor for CRS with statistical significance in both studied groups: “summer” and “winter” (*p* = 0.0001 and *p* = 0.00020, respectively)
Nandyal et al. [25]	2024	India	prospective/observational	70	18 to 70 years	42	28	CT	a/c/s	x	The study was conducted on 70 patients with chronic rhinosinusitis using CT scans to identify anatomical variants.	Maxillary CRS is associated with the presence of HC (*p* < 0.05)

**Table 3:** Study quality assessment.

	Azila et al. (2011) [31]	Ali et al. (2017) [26]	Kamdi et al. (2018) [27]	Devaraja et al. (2019) [28]	Özcan et al. (2021) [29]	Hui et al. (2022) [30]	Nedelcu et al. (2024) [12]	Nandyal et al. (2024) [25]
Were the criteria for inclusion in the sample clearly defined?	x	x	x	0	x	x	x	x
Were the study subjects and the setting described in detail?	0	x	x	x	x	x	x	x
Was the exposure measured in a valid and reliable way?	x	x	x	x	x	x	x	x
Were objective, standard criteria used for measurement of the condition?	0	x	x	0	x	x	x	x
Were confounding factors identified?	x	x	x	x	x	x	x	0
Were strategies to deal with confounding factors stated?	0	0	x	0	x	0	x	0
Were the outcomes measured in a valid and reliable way?	x	x	x	x	x	x	x	x
Was appropriate statistical analysis used?	x	x	x	x	x	x	x	x
Total	8	10	11	8	11	10	11	9

## Data Availability

All relevant data are contained within the manuscript. The raw data supporting the conclusions of this article will be made available by the authors on request.

## Abbreviations

The following abbreviations are used in this manuscript:

HCs	Haller cells
CRS	Chronic rhinosinusitis
CT	Computed Tomography
MDCT	Multi-detector computed tomography
CBCT	Cone-Beam Computed Tomography
ARS	Acute rhinosinusitis
CRSwNP	Chronic rhinosinusitis with nasal polyps
CRSsNP	Chronic rhinosinusitis without nasal polyps
AERD	Exacerbated respiratory disease
MPR	Multiplanar reconstruction
OR	Odds ratio
CI	Confidence interval
JBI	Joanna Briggs Institute
